# Visuomotor control of intermittent circular tracking movements with visually guided orbits in 3D VR environment

**DOI:** 10.1371/journal.pone.0251371

**Published:** 2021-05-27

**Authors:** Woong Choi, Naoki Yanagihara, Liang Li, Jaehyo Kim, Jongho Lee

**Affiliations:** 1 Department of Information and Computer Engineering, National Institute of Technology, Gunma College, Maebashi, Japan; 2 Department of Computer Science, University of Tsukuba, Tsukuba, Japan; 3 College of Information Science and Engineering, Ristumeikan University, Kusatsu, Japan; 4 Department of Mechanical and Control Engineering, Handong Global University, Pohang, Republic of Korea; 5 Department of Clinical Engineering, Komatsu University, Komatsu, Japan; Washington University in Saint Louis School of Medicine, UNITED STATES

## Abstract

The analysis of visually guided tracking movements is important to the understanding of imitation exercises and movements carried out using the human visuomotor control system. In this study, we analyzed the characteristics of visuomotor control in the intermittent performance of circular tracking movements by applying a system that can differentiate between the conditions of invisible and visible orbits and visible and invisible target phases implemented in a 3D VR space. By applying visuomotor control based on velocity control, our study participants were able to track objects with visible orbits with a precision of approximately 1.25 times greater than they could track objects with invisible orbits. We confirmed that position information is an important parameter related to intermittent motion at low speeds (below 0.5 Hz) and that tracked target velocity information could be obtained more precisely than position information at speeds above 0.5 Hz. Our results revealed that the feedforward (FF) control corresponding to velocity was delayed under the visible-orbit condition at speeds over 0.5 Hz, suggesting that, in carrying out imitation exercises and movements, the use of visually presented 3D guides can interfere with exercise learning and, therefore, that the effects of their use should be carefully considered.

## Introduction

We use countless body movements to observe, hold, and move objects in our daily lives. In particular, the visually guided tracking of movements is an important function in learning or imitating movement [1–5].

Research on visually guided movement tracking has focused on tasks in which the trajectories of visually guided targets are tracked along one-dimensional (1D) straight lines or on two-dimensional (2D) planes through various joint movements in a three-dimensional (3D) space [6–16]. For example, Miall *et al*. (1986, 1988, 1993) examined the task of tracking a visually guided target with a 1D sinusoidal trajectory using the multi-joint motion of an arm in a 3D space. Tests in which monkeys and humans carried out this task revealed that the control parameters differed depending on the periodicity of the target orbit on the 1D tracking space. Beppu *et al*. (1984, 1987) performed tests in which patients with cerebellar disease and control patients carried out a tracking task using an elbow joint motion with one degree of freedom under the visual guidance of a 1D ramp trajectory. Their results revealed parameters that could quantitatively evaluate the severity of cerebellar disease in patients.

Circular tracking movements have periodic tracking characteristics similar to those of 1D sinusoidal tracking movements. Unlike 1D tracking, however, circular tracking enables the examination of constant-velocity continuous movement on a 2D plane [12–19]. Several studies have examined the tracking of targets with visually guided trajectories on a 2D plane using a stylus-equipped tablet, a 2D tracer (i.e., computer mouse), and two-degree-of-manipulandum arm and wrist movements. In our previous research, we analyzed the characteristics of visuomotor control using 2D circular tracking movements in a polar coordinate system in which the feature parameters theta and omega measured the precisions of position and velocity control, respectively [12]. We confirmed that these parameters could be used to analyze the characteristics of visuomotor control in carrying out circular tracking movements in a 3D VR space under monocular and binocular vision conditions [23–24].

Intermittent circular tracking movements can be used to study the characteristics of motor control under feedback (FB) and feedforward (FF) visuomotor control [8, 10–14]. In systems in which a target and tracer are visible on some sections of a circular tracking path, position correction using FB control can be performed to reduce the difference between the two. Similarly, FF-based prediction control for tracking targets along trajectory sections in which they are invisible have been carried out [12, 14]. Previous studies on humans compared the effects of the visible and invisible phases of 2D circular tracking movement on the FB and FF controls [12, 14]. However, in previous studies the orbit of the target was displayed to make it easier for the subject to perform the experiment. In these studies, the FF control in the invisible phase of the target orbit was implemented via position correction control obtained from visual information regarding the orbit. Furthermore, previous studies did not consider the effect of the quadrant on circular tracking movement in analyzing the data; instead, data were adopted from different quadrant regions based on the visible and invisible phases of the 2D circular tracking movement.

Given the existing literature background, we pose the following challenging question: What are the differences between the characteristics of visuomotor control in visible and invisible target phases along visible and invisible target orbits during 3D target-tracking movements? To address this, we examined data obtained from the quadrant regions corresponding to the visible and invisible phases of a 3D intermittent circular tracking movement.

Thus, our study investigated the characteristics of visuomotor control in carrying out intermittent circular movement by implementing a system that presented the conditions of visible and invisible circular orbits in a 3D VR space during visible and invisible target phases. As controlling the position and speed of movement is closely related to the functioning of the human cerebellum, which controls body movement [20–22], we utilized position and velocity parameters measured in polar coordinates to analyze visuomotor control [12, 23, 24]. Finally, we quantitatively analyzed the characteristic of the FB and FF controls based on the changes in target speed between the visible and invisible circular orbit conditions on the same quadrant regions.

## Materials and methods

The subjects recruited for the study comprised 18 men and one woman with a mean age of 20 ± 0.71 years (Table 1). All subjects had normal or corrected-to-normal vision with a binocular vision greater than 0.7. The visual acuity of each subject was determined based on the results of their health examination. None of the subjects had previously participated in similar studies. All subjects provided written informed consent prior to participation. All experiments were conducted in accordance with the relevant guidelines and regulations following a protocol approved by the ethics committee of the National Institute of Technology, Gunma College.

**Table 1:** Subject information: Age, sex, and dominant hand.

*Subject no*.	*Age*	*Sex*	*Dominant* *hand*
1	19	M	R
2	20	M	R
3	21	M	L
4	21	M	R
5	20	M	R
6	20	M	R
7	19	M	R
8	20	M	R
9	20	M	R
10	20	M	R
11	19	M	R
12	20	M	R
13	20	M	R
14	19	F	R
15	19	M	R
16	20	M	R
17	20	M	L
18	21	M	L
19	21	M	R

**Fig 1:**
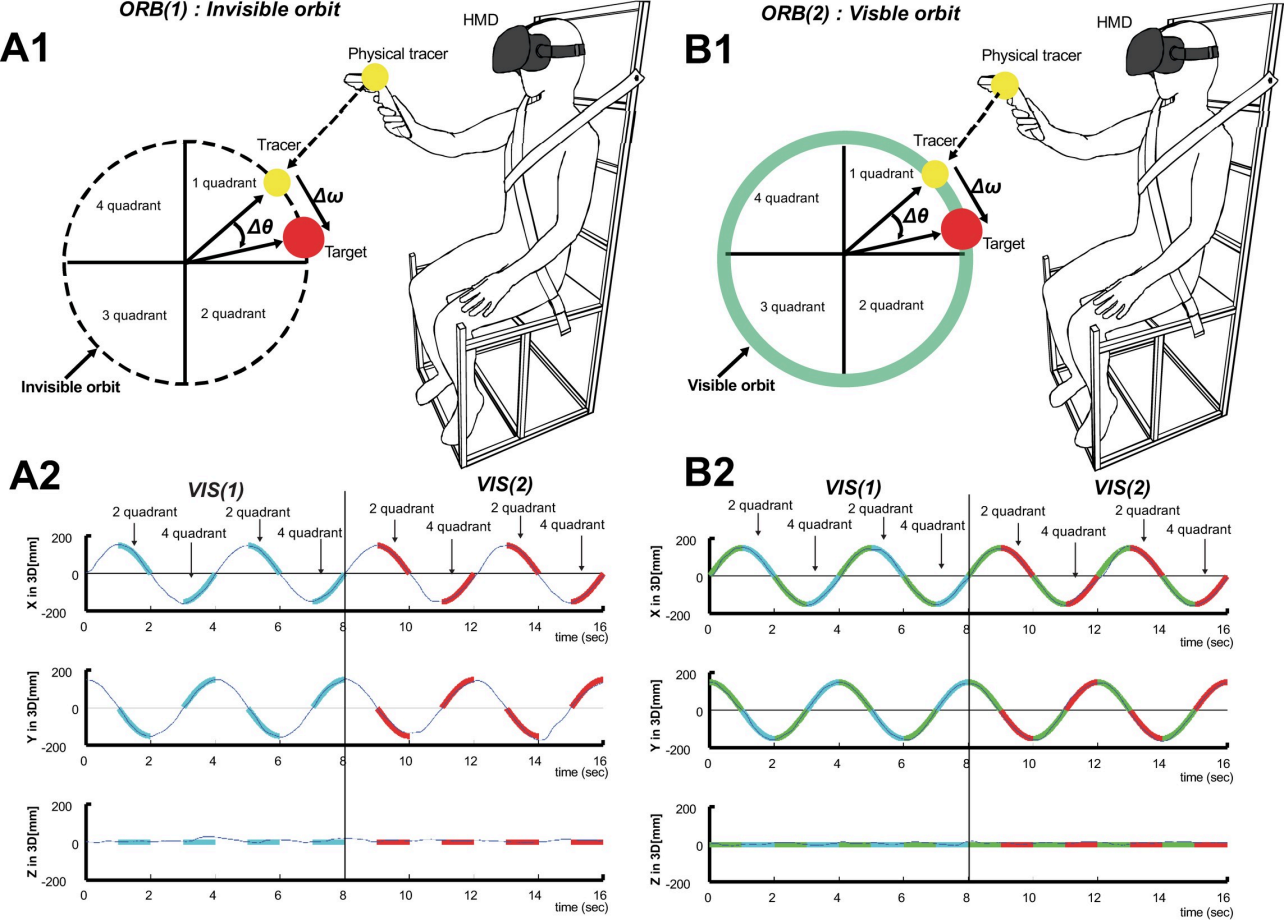
Experimental procedure. *VIS(1)*: visible target and tracer, *VIS(2)*: only visible tracer. *ORB(1)*: invisible circular orbit, *ORB(2)*: visible circular orbit. **(A1)** Schematic of circular tracking experiment for *ORB(1)*. Under *ORB(1)*, the target path is not visible to the subjects during the experiment. **(B1)** Schematic of circular tracking experiment for *ORB(2)*. Under *ORB(2)*, the target path is visible to the subject. The green lines indicate the target path in the 3D VR space. The insets on the left-hand sides of (A1) and (B1) show how the two outcome measures (*Δθ*, *Δω*) were derived from the path data of the target (or tracer) under each condition. In both (A1) and (B1), the target rotates clockwise at a constant speed, moving from quadrants 1 to 4 in order. **(A2)** Typical trial data showing tracer path (black line) versus time at a speed of 0.25 Hz on three axes. **(B2)** Typical trial data showing target (green line) and tracer paths (black line) versus time at a speed of 0.25 Hz on three axes. The three graphs in (A2) and (B2) show the path of the tracer as seen from the X-, Y-, and Z-axes, respectively. In the graphs, regions *VIS*(1) and *VIS*(2) are indicated in cyan and red, respectively.

Each subject was asked to conduct intermittent 3D circular tracking tasks under visual guidance in which a tracer was used to track a virtual target in a 3D VR space (Fig 1) [23–25]. In carrying out the tasks, the position of the tracer was synchronized with the hand movements of the subject. During the experiment, the target was moved continuously along visible and invisible circular orbits with radii of 15 cm, as shown in Fig 1(A1) and 1(B1), respectively. The insets on the left of (A1) and (B1) show how the two outcome measures (*Δθ*, *Δω*) were derived from the path data of the target (or tracer) under each condition. In (A1) and (B1), the target rotates clockwise at a constant speed and moves from quadrants 1 to 4 in order. (A2) and (B2) show typical trials of the tracer path (black line), which is plotted versus time at a speed of 0.25 Hz on the X-, Y-, and Z-axes, respectively. On the graphs, the regions in which the visible target and tracer (*VIS(1)*) and only the visible tracer (*VIS(2)*) are displayed are indicated in cyan and red, respectively. Data from the second and fourth quadrant regions corresponding to *VIS(1)* and *VIS(2)*, respectively, were used to assess the 3D intermittent circular tracking movement.

We performed an additional examination to evaluate the stereo acuity of the subjects in the VR space. After calibrating the display position of the target, each participant was asked to confirm verbally whether they could appropriately perceive it; if not, the inter-ocular distance was adjusted accordingly. The stereo acuity of the participant was then evaluated by performing a task in which the tracer was placed at the center of a 3D target. Each subject who successfully performed this task four times or more in five attempts was selected for further research.

### Experimental task

The experiments were performed in a VR space (Fig 1). In each experiment, the subject was seated in a custom-built chair with a head-mounted display placed on their head while holding a controller in their dominant hand. The initial position of the target was then calibrated for each subject. The target was rotated at 0.25, 0.5, or 0.75 Hz along a visible or invisible orbit after a countdown of 3 s accompanied by sound effects, as shown in Fig 1(A1) and 1(B1). During the countdown, the subject was asked to move the tracer to the target position and then perform a circular tracking movement. As shown in Fig 1, in each trial the target was stopped after four revolutions. In total, there were four trials in which the target rotated in the frontal plane over an invisible circular orbit (*ORB(1)*) and a visible circular orbit (*ORB(2)*). Thus, for each subject 192 revolutions were carried out in total (4 revolutions × 4 trials × 3 speeds × 2 phases × 2 orbits). In all, there were 12 experimental conditions (3 speeds × 2 phases × 2 orbits). The first trial under each setting was discarded from the analysis to account for the adjustments required on the part of the subjects with respect to the experimental protocol. After the movements made by each subject were tracked under condition *VIS(1)*, they were tracked under the visible tracer condition *VIS(2)* because the circular tracking model was generated by the efference copy in the brain.

### Data analysis

To measure data, polar instead of Cartesian coordinates were used, with the angular displacement “θ” and angular velocity “ω” in polar coordinates measured for data analysis (see Fig 1). Because the second and fourth quadrants of the tracking circle had phases of *VIS(2)* in the experimental task, we used data extracted from these quadrants under each experimental condition, as shown in Fig 1(A2) and 1(B2). The data obtained from the first trial under each condition were discarded from the analysis.

The absolute value of the difference in angular displacement between the target and the tracer can be defined as

$$\Delta \theta =|\theta_{tracer}-\theta_{target}|,$$ (1)

similarly, *Δω*, the absolute value of the difference in angular velocity between the target and tracer, can be expressed as

$$\Delta \omega =|\omega_{tracer}-\omega_{target}|.$$ (2)

In this study, we investigated the differences in the parameters *Δθ* and *Δω* between the circular tracking movements on the frontal plane.

To analyze the differences in circular tracking movements in terms of Δ*θ* and Δ*ω*, we carried out a three-way repeated measure analysis of variance (ANOVA) based on the phase factor (at two levels, namely *VIS(1)*: visible target and tracer, and *VIS(2)*: only visible tracer), the speed factor (at three levels, namely *V(1)*: 0.25 Hz (n = 19), *V(2)*: 0.5 Hz (n = 19), and *V(3)*: 0.75 Hz (n = 19)), and the orbit factor (at two levels, namely *ORB(1)*: invisible circular orbit, and *ORB(2)*: visible circular orbit). Table 2 summarizes the factors and levels used in the statistical analysis.

**Table 2:** Summary of the factors and levels related to the statistical analysis.

*Factors*	*Levels*	*Condition*
Phase	*VIS(1)*	visible target and visible tracer
	*VIS(2)*	only visible tracer, invisible target
Orbit	*ORB(1)*	invisible orbit
	*ORB(2)*	visible orbit
Speed	*V(1)*	target speed of 0.25 Hz
	*V(2)*	target speed of 0.50 Hz
	*V(3)*	target speed of 0.75 Hz

The main effects and interactions between the phase, speed, and orbit factors with respect to *Δθ* and *Δω* were assessed using the repeated measures function in the IBM SPSS Statistics software [26]. A post-hoc test was conducted through pairwise comparisons of the Bonferroni correction. Except where noted, we described the data in terms of the mean (*M*), standard error (*SE*), and standard deviation (*SD*). We considered comparisons yielding *p* < 0.05 to be statistically significant and those yielding p < 0.01 to be highly statistically significant. These methods and statistical analyses were used to produce the data listed in S1 and S2 Tables.

Using the G*Power software, we performed a priori power analysis to determine the minimum required sample size for repeated measures and within-between interaction ANOVA [27]. For an effect size of 0.25, alpha = 0.05, number of groups = 12, power = 0.80, number of measurements = 3, and non-sphericity correction = 1, the analysis revealed that 17 participants were required to detect with an actual power of 0.8. To meet this minimum sample size, we tested 19 subjects.

## Results

We studied the characteristics of visuomotor control using the measured *Δθ* and *Δω* values obtained under each phase and orbit level during the intermittent 3D target-tracking movements.

### Effect of phase and orbit factors on visuomotor control during intermittent 3D target-tracking movements in terms of Δθ

**Fig 2:**
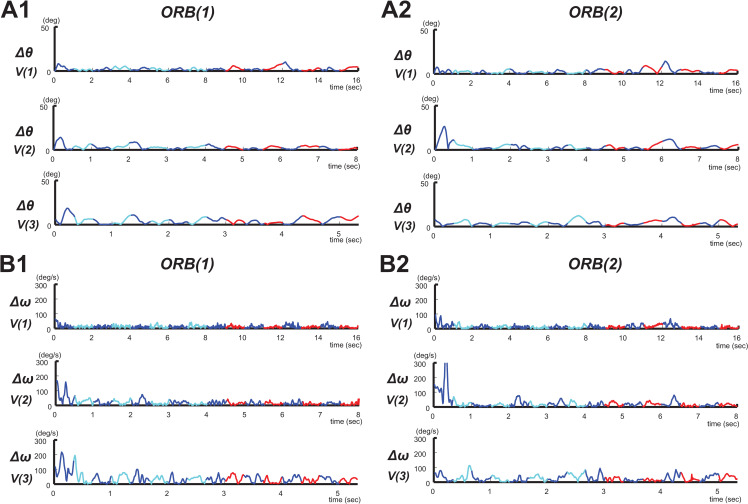
Typical examples of circular tracking movements at speeds of 0.25, 0.5, and 0.75 Hz. *V(1)*: 0.25 Hz (n = 19), *V(2)*: 0.5 Hz (n = 19), and *V(3)*: 0.75 Hz (n = 19). (**A1**) Absolute values of Δ*θ* under *ORB(1)* and (**A2**) under *ORB(2)*. (**B1**) Absolute values of Δ*ω* under *ORB(1)* and (**A2**) under *ORB(2)*. In the graphs, the regions of *VIS(1)* and *VIS(2)* are indicated in cyan and red, respectively.

Fig 2(A1) and 2(A2) show the values of *Δθ* for each orbit and phase level at the three target speeds (0.25, 0.5, and 0.75 Hz).

The values of Δθ were assessed to investigate the differences in the position angle between VIS(1) and VIS(2) at each orbit level and target speed. A three-way repeated-measures ANOVA (orbit × phase × speed) revealed that there was a significant second-order interaction between the phase, speed, and orbit (F (1.368,24.619) = 5.349, p = 0.021, partial η^2^ = 0.229; Item A in S1 Table). There was also a significant effect in terms of speed *(F* (2, 36) = 64.111, *p* = 0.000, *partial η*^*2*^ = 0.781), indicating that the speed factor affected *Δθ* in the intermittent circular tracking movement results. In addition, the interaction between the orbit and phase factors at each target speed affected the performance of *Δθ* in evaluating the precision of the position control.

The orbit levels *ORB(1)* (*M* = 5.9°, *SE* = 0.385°) and *ORB(2)* (*M* = 5.88°, *SE* = 0.343°) performed nearly identically in synchronizing the position angle of the target and tracer (*F* (1,18) = 0.005, *p* = 0.944, *partial η*^*2*^ = 0). By contrast, the speed factor was found to increase the level of speed (0.25 Hz: *M* = 3.67°, *SE* = 0.206°; 0.5 Hz: *M* = 5.302°, *SE* = 0.324°;0.75 Hz: *M* = 8.709°, *SE* = 0.653°) (*F* (1.1,19.78) = 64.11, *p* = 0, *partial η*^*2*^ = 0.78).

**Fig 3:**
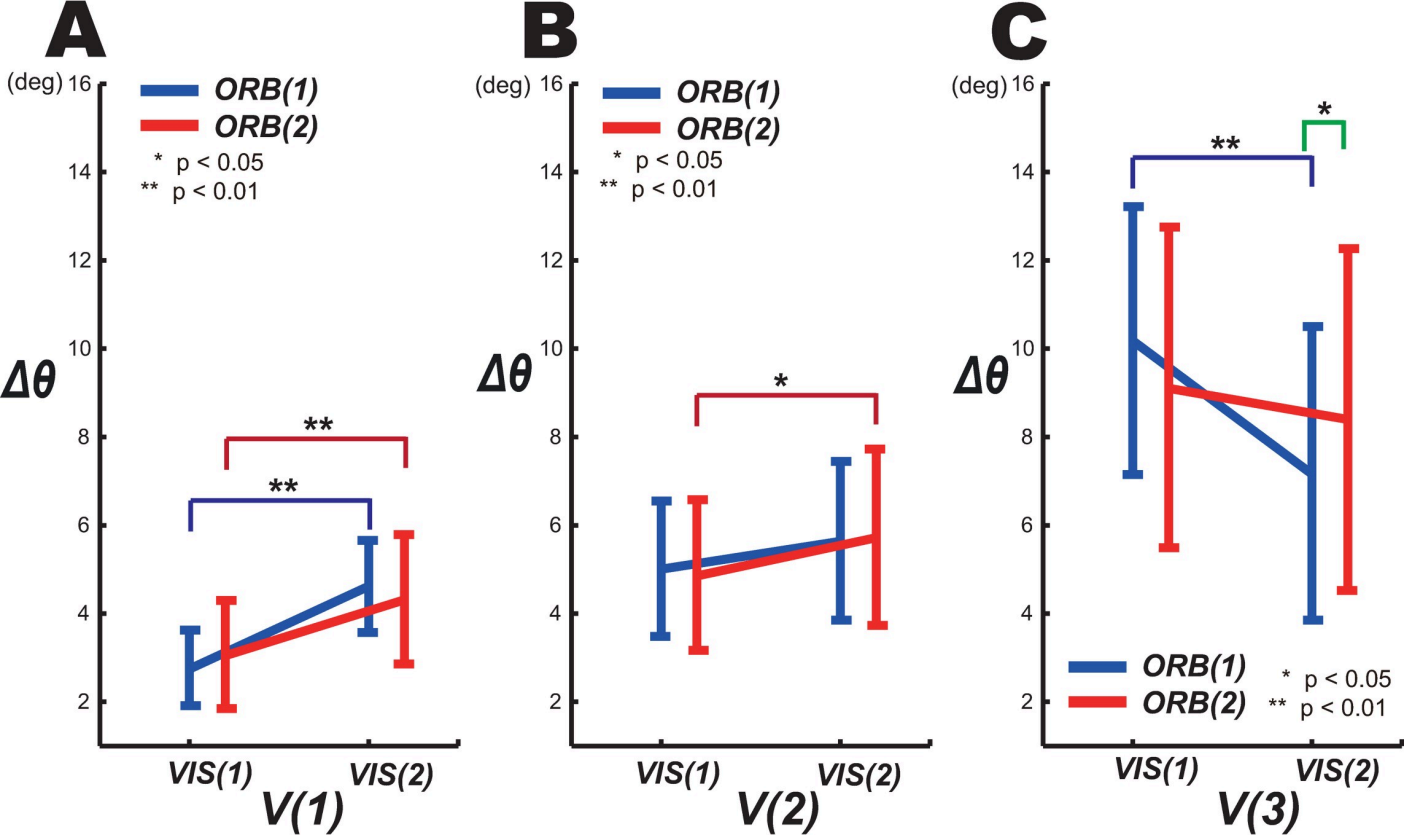
Effect of visuomotor control on *Δθ*. For each *ORB* level, effects of *VIS* on *Δθ* are shown at (A) 0.25, (B) 0.5, and (C) 0.75 Hz.

We next investigated effect of the phase level on the position angle for *ORB(1)* and *ORB(2)* (Fig 3). A two-way repeated-measures ANOVA (orbit × phase) revealed that there was significant interaction between phase and orbit (Item F in S1 Table), although the simple effect of the orbit factor on *Δθ* indicated a significant difference by orbit factor at *VIS*(2) (*F* (1,18) = 5.532, *p* = 0.03, *partial η*^*2*^ = 0.235; Item G in S1 Table). The *Δθ* of *ORB(1)* for *VIS*(2) was less than that of *ORB(2)* at 0.75 Hz (Fig 3(C)).

The differences in *Δθ* between phase levels at each orbit level and target speed were then examined. Fig 3 shows the results of a pairwise comparison (Bonferroni correction) in terms of *Δθ* (Item H in S1 Table).

Under both *ORB(1)* and *ORB(2)*, there were significant differences between the phase levels at 0.25 Hz. Because *VIS(1)* was performed under the FB control, which depends on the visual information of the target, at the low speed of 0.25 Hz, its results differed significantly from those of *VIS(2)*, which was performed without visual information of the target and therefore produced a larger error in position than *VIS(1)*. At 0.5 Hz, there was a significant difference between *VIS(1)* and *VIS(2)* under the visible-orbit condition *ORB(2)*. At 0.75 Hz, the *Δθ* for *VIS(2)* tended to be lower than that for *VIS(1)*; in particular, there was no significant difference between *VIS(1)* and *VIS(2)* under *ORB(2)* at 0.75 Hz. The results indicated that, in controlling the position angle, the orbit factor did not delay the start of the FF control at speeds greater 0.5 Hz. In addition, the *Δθ* of the FF control was less than that of the FB control at 0.75 Hz (Fig 3(C)) and the FB control of the position angle in 3D circular tracking movement was superior to the FF control at speeds below 0.5 Hz.

### Effect of phase and orbit factors on visuomotor control during intermittent 3D target-tracking movements in terms of *Δω*

Fig 2(B1) and 2(B2) show the values of *Δω* obtained under various orbit and phase levels at the three target speeds (0.25, 0.5, and 0.75 Hz).

We evaluated Δ*ω* to compare the velocity control accuracies under the *VIS(1)* and *VIS(2)* levels at each orbit level and target speed. A three-way repeated-measures ANOVA (orbit × phase × speed) revealed a significant second-order interaction between the phase, speed, and orbit (*F* (1.423,25.61) = 11.86, *p* = 0.001, *partial η*^*2*^ = 0.397; Item A in S2 Table). Furthermore, significant effects were observed in terms of orbit (*F* (1, 18) = 29.61, *p* = 0.000, *partial η*^*2*^ = 0.622) or phase (*F* (1, 18) = 78.94, *p* = 0.000, *partial η*^*2*^ = 0.814) and speed (*F* (1.194, 21.49) = 247.624, *p* = 0.000, *partial η*^*2*^ = 0.932). This indicates that, under intermittent circular tracking, *Δω* was affected by the orbit, phase, and speed factors and that the interactions between these three factors affected the performance of *Δω* in evaluating the velocity-control precision of the intermittent circular tracking movements.

The participants were, overall, more accurate in velocity control under *ORB(2)* (*M* = 26.13° s^-1^, *SE* = 0.88° s^-1^) than under *ORB(1)* (*M* = 32.61° s^-1^, *SE* = 1.67° s^-1^) when synchronizing the angular velocities of the target and tracer (*F* (1,18) = 29.61, *p* = 0, *partial η*^*2*^ = 0.62). The tracking movement was, in general, more accurate in carrying out velocity control under *VIS(1)* (*M* = 25.854° s^-1^, *SE* = 1.072° s^-1^) than under *VIS(2)* (*M* = 32.887° s^-1^, *SE* = 1.424° s^-1^) (*F* (1,18) = 78.94, *p* = 0, *partial η*^*2*^ = 0.81). Moreover, the speed factor was found to increase the level of speed (0.25 Hz: *M* = 14.4° s^-1^, *SE* = 0.585° s^-1^; 0.5 Hz: *M* = 26.3° s^-1^, *SE* = 1.09° s^-1^;0.75 Hz: *M* = 47.42° s^-1^, *SE* = 2.243° s^-1^) (*F* (1.19,21.49) = 247.62, *p* = 0, *partial η*^*2*^ = 0.932).

**Fig 4:**
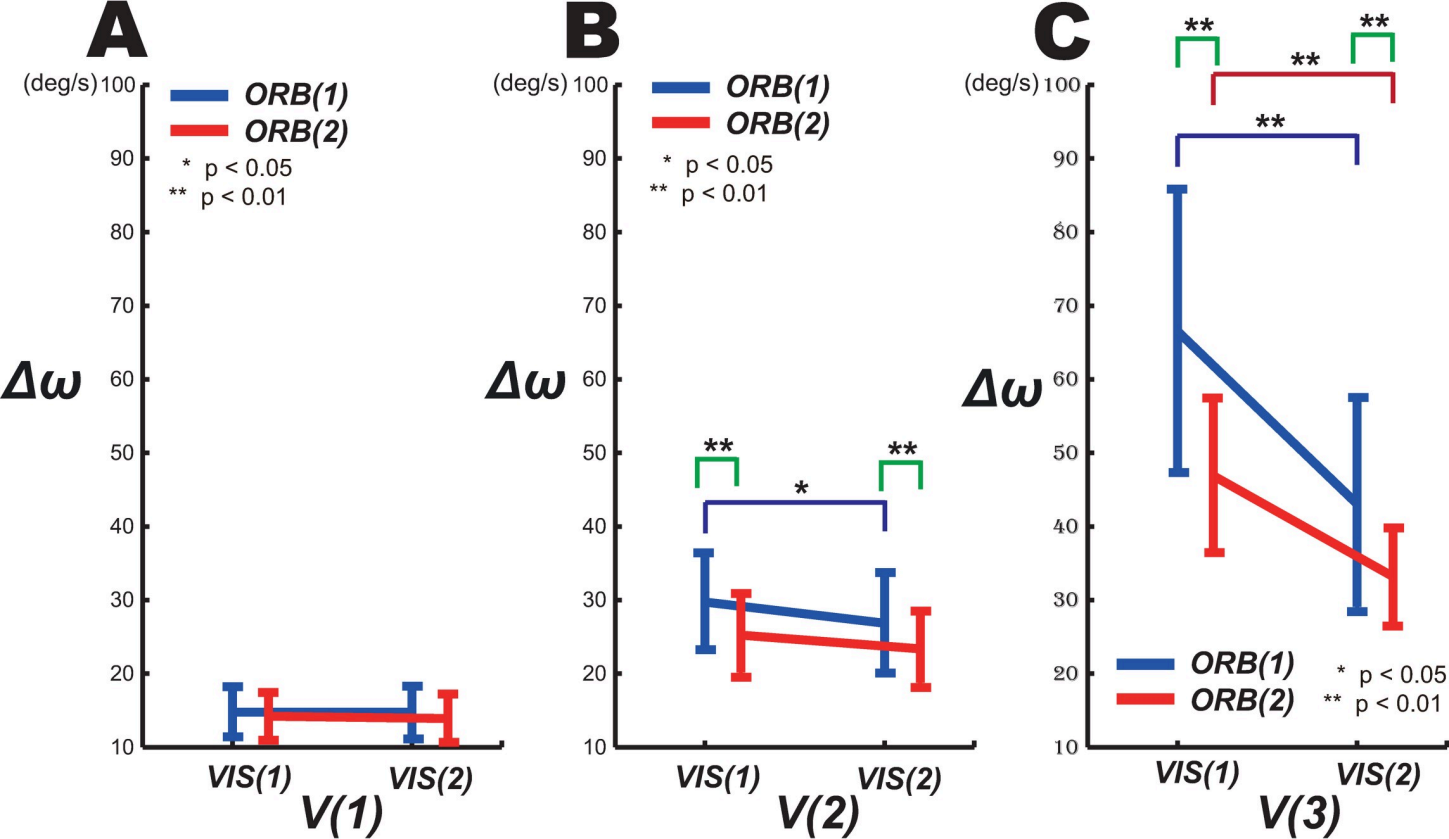
Effect of visuomotor control on *Δ*ω. For each *ORB* level, effects of *VIS* on *Δ*ω are shown at (A) 0.25, (B) 0.5, and (C) 0.75 Hz.

We next evaluated the differences in velocity control accuracy by phase level under *ORB(1)* and *ORB(2)* (Fig 4). A two-way repeated-measures ANOVA (orbit × phase) revealed that there was significant interaction between phase and orbit (Item F in S2 Table). In addition, the simple effects on *Δω* differed significantly by orbit level (Item G in S2 Table). Furthermore, there were the significant results of pairwise comparisons (Bonferroni correction) (Item E in S2 Table) at 0.5 Hz. Above 0.5 Hz, the velocity control accuracy of 3D circular tracking was significantly improved under *ORB(2)* as shown in Fig (4B) and (4C).

The effects on *Δω* of orbit level and target speed at both phase levels were then examined. Fig 4 shows the results of pairwise comparisons (Bonferroni correction) between the *Δω* results obtained under *VIS(1)* and *VIS(2)* for the *ORB(1)* and *ORB(2)* conditions at the three target speeds. The differences in *Δω* by phase level were found to be statistically significant on Item F in S2 Table.

This result suggests that the participants were generally more accurate at velocity control under the *ORB(2)* (*M* = 26.13° s^-1^, *SE* = 0.88° s^-1^) condition than under the *ORB(1)* (*M* = 32.61° s^-1^, *SE* = 1.67° s^-1^) condition when synchronizing the angular velocities of the target and tracer at speeds above 0.5 Hz (*F* (1,18) = 29.61, *p* = 0, *partial η*^*2*^ = 0.62; Item A of S2 Table). The accuracy in terms of angular velocity under *ORB(2)* was approximately 1.25 times higher than that under *ORB(1)*. Under *ORB(1)*, FF control with increasing target speed began above 0.5 Hz whereas, under *ORB(2)*, FF control began at 0.75 Hz, a delay in FF control onset that we attribute to the presence of a visible orbit. Furthermore, at speeds above 0.5 Hz the values of *Δω* obtained under *VIS(2)* decreased relative to those under *VIS(1)*.

## Discussion

In this study, we quantitatively evaluated the motor control characteristics of the intermittent circular tracking movements of participants tracking a target orbit in a 3D VR space. Our analysis of the spatiotemporal relationship between the visible and invisible target phases during the 3D target-tracking process revealed that the visible orbit condition delayed the onset of the FF control of the kinematic parameters, i.e., the position and velocity, during the intermittent circular tracking movement. By using visuomotor control based on velocity control, the subjects performed tracking with a precision of approximately 1.25 greater under the visible orbit condition than under the invisible condition.

In the following, we discuss three issues: (1) the differences between the visible and invisible target phases in terms of the characteristics of visuomotor control used to carry out intermittent 3D target tracking movement; (2) the differences between the visible and invisible orbit phases in terms of these visuomotor control characteristics; and (3) the limitations and future application of the proposed system.

### Differences between visuomotor control characteristics used to carry out intermittent 3D target tracking under visible and invisible target phases

To enact visuomotor control in carrying out intermittent circular tracking movements, tracking movements under the target-visible phase (VIS(1) in Figures) primarily involve the application of FB control, including control under the forward model involving visual and proprioceptive information. By contrast, tracking movements under the target-invisible phase (VIS(2) in Figures) are more dependent on FF control applying the inverse internal model of target motion in the central nervous system (CNS) [8, 10–14]. In other words, visuomotor control in tracking movements to a moving target must consider two problems: the sensory feedback noise and delay in the motor system (i.e., 100–200-ms sensorimotor delay [28–31]), and the environmental target motions (i.e., the position and velocity information corresponding to target motion). To address the motor system problem, recent studies have applied forward internal models that can predict the sensory consequences of a motor command by using its efferent copy [32]. The theory assumes that the CNS estimates the position of the hand by combining the predictions made by the forward model using the efference copy with actual sensory feedback to perform accurate goal-directed movements. Furthermore, during periodic target tracking such as that used in a circular tracking task, the CNS acquires information (i.e., position and velocity) on target motion by applying an inverse model in which the hand position is compared with the desired target state based on feedback error learning [33, 34]. The results of this study suggest that FB control under the VIS(1) target-visible phase prediction is governed more by the prospective forward model than by the retrospective inverse model as the former allows for accurate comparison of the states (position and velocity) of the target and tracer during on-line target tracking movement [35–38]. By contrast, under the target-invisible VIS(2) phase the significance of the FF controller using the inverse model exceeds that of the FB controller using the forward mode because comparison of the tracer and target states is impossible as all target visual information has been removed. In other words, in the VIS(2) phase the overall motor commands to enact arm movements are primarily defined by FF using the inverse model [33, 34].

Previous studies qualitatively demonstrated the differences in the frequency components or the smoothness of the trajectory between the FB-dominant VIS(1) and FF-dominant VIS(2) phases through the enhanced and reduced intermittencies obtained, respectively, when using FB and FF control [8, 10]. By contrast, this study examined relative changes in the control parameters (i.e., the angular error (Δθ) and angular velocity error (Δω) as indices of position-control and velocity-control precision, respectively) between the FB-dominant VIS(1) and FF-dominant VIS(2) phases, revealing quantitative changes in control strategy (i.e., the use of position and velocity controls) in terms of, for example, which parameter was more accurately considered under the FB and FF controls. In other words, we analyzed how FB and FF control mutually interact with these control parameters in carrying out intermittent circular tracking movements. For example, the increase in Δθ (representing the position-control precision) from VIS(1) to VIS(2) at velocities below 0.5 Hz (Fig 3) revealed that positional information was the more critical parameter for FB control in the target-visible phase of slow target tracking movement. Similarly, the decrease in Δω (representing the velocity-control precision) from VIS(1) to VIS(2) (Fig 4) revealed that velocity information was more important in FF control in the target-invisible phase.

### Differences between visuomotor control characteristics used to carry out intermittent 3D target tracking on visible and invisible orbits

In previous studies, the differences in the effect of orbit visibility between the *ORB(1)* and *ORB(2)* phases on 3D circular tracking movement were not quantitatively analyzed; instead, they were simply displayed to enable easier 2D circular tracking during the invisible target phase [12, 14]. In this study, we quantitatively analyzed the differences between *ORB(1)* and *ORB(2)* under 3D circular tracking.

Previous studies reported that, when the target disappeared during the circular tracking movement, *Δω* was reduced and predictive FF control was performed [12]. The results of this study revealed significantly that, at target speeds of 0.5 Hz and above, the *Δω* values under *VIS(2)* were lower than those under *VIS(1)* under the invisible-orbit *ORB(1)* condition. Furthermore, at 0.5 Hz the FF control corresponding to the velocity was delayed by the presence of a visible orbit; in other words, at target speeds of 0.75 Hz or above there was a significant difference between the orbit conditions in that the Δω of VIS(2) was lower than that of VIS(1) under the orbit-visible ORB(2) condition.

It is widely known that the cerebellum detects and corrects for errors in the on-line control of movement. This error processing is a central component of cerebellar functions including motor learning [39, 40] and the construction of internal models [32–34]. Error signals in the cerebellum are generally believed to be transmitted via climbing fiber input. In other words, Purkinje cell complex spike discharge is associated with errors during eye [41, 42] and arm movements [39, 43, 44]. In the VIS(1) phase examined in this study, the FB controller detected and corrected errors based on target and tracer visual information during the tracking movement. When the target visual information was eliminated under the VIS(2) phase, the FB controller failed to detect these errors and, as a result, the FF controller was primarily used instead to encode circular tracking movement. Under the visible-orbit condition ORB(2), the presence of visual orbit information appeared to induce the FB controller to attempt to detect the errors between the visual tracer and the memorized target during the target-invisible VIS(2) phase using the cerebellum. Consequently, at high speeds the FF control might be delayed (impaired) by the visibility of an orbit. In other words, through a comparison of the visible and invisible orbit conditions (Figs 3 and 4) we revealed that the visual presentation of 3D guides might interfere with the learning processing that is a central component of cerebellar functions such as motor learning and internal modeling.

As the target speed increases, the visuomotor control of the temporal space depends increasingly on FF control based on velocity in place of FB control [6, 12, 24]. The benefits of tracking using a guideline at slow target speeds and ages were reported in [45]. Our results revealed that, at target speeds above 0.5 Hz, limb FF control was superior under intermittent circular tracking movement regardless of the orbit level (Fig 4).

Furthermore, we found that, consistent with a previous study [12], under the *ORB(2)* condition *Δθ* increased under *VIS (2)* at *V(1)* and *V(2)*. This result indicates the position control of circular tracking movement is difficult in the absence of visual target information in 3D space. At *V(3)*, FB control was more difficult than FF control even when the target information was present. There were significant differences between the results for *V(1)* and *V(3)* under *ORB(1)*, when the orbit was not visible. At the lowest-speed condition, *V(1)*, the *Δθ* under *VIS(1)* was less than that under *VIS(2)*, whereas at the highest-speed condition, *V(3)*, the *Δθ* under *VIS(2)* was less than that under *VIS(1)*.

These results indicate that, when the speed is less than 0.5 Hz, the FB control, which depends on the visual information of the target, dominates; by contrast, when the speed is greater than 0.5 Hz, the FF control based on the prediction of the target dominates.

A previous study reported that the omega parameter is an important component of intermittent movement based on changes in speed [12]. In this study, we found that, at speeds of less than 0.5 Hz, the position-related theta parameter is an important indicator of intermittent, speed-based motion during the 3D circular tracking movement. Moreover, the velocity information of the tracking target could be more precisely obtained than its position information and could be used as a control model by participants in carrying out intermittent circular tracking movement at speeds greater than 0.5 Hz.

The FF control is necessary to compensate for the delays in sensory information [24, 39] that are present in all stages of the sensorimotor system from reception of afferent sensory information to the response of muscles to efferent motor commands [28]. The results of this study revealed that the FF control corresponding to velocity was delayed speeds of 0.5 Hz when the orbit was visible. This indicates that a visually presented orbit can delay the onset of the FF control and suggests that the use of visually presented guides in imitation exercises and movements could interfere with exercise learning.

### Future applications and limitations of the proposed system

In this study, we quantitatively analyzed the effects of using a target with a constant speed and direction experimentally. In future studies, we plan to analyze the effects of target irregularity in terms of the direction and speed of intermittent circular tracking movement by adding conditions in which the directional motion of the target changes irregularly or the speed is not constant.

Individuals with disabilities or damage to the visuomotor system as a result of illness or accidents can have difficulty in working with their hands in carrying out tracking tasks. The system proposed in this study can help in the setting up of spatiotemporal environments for the tracking of targets through the use of visually presented orbits. In particular, the level of visuomotor movement associated with the cerebellum can be quantitatively analyzed through the presentation of intermittent movement.

## Conclusions

In this study, we quantitatively analyzed the visuomotor control characteristics of intermittent circular tracking movements displayed as target orbits in 3D VR space. Our research examined relative changes in the use of control parameters (i.e., Δθ and Δω as indices of position and velocity control precision, respectively) between FB- and FF-dominant conditions indicating quantitative changes in control strategy (i.e., position and velocity controls). We found that, because they could apply visuomotor control based on velocity control, the tested individuals improved their precision of target tracking by a factor of approximately 1.25 when the tracking orbit was visible. It was shown that, at the low-speed condition below 0.5 Hz, positional information is a more critical parameter for FB control in the target-visible phase and that, at the high-speed condition above 0.5 Hz, velocity information is more relevant to FF control under the target-invisible phase. Our results revealed that the FF control corresponding to velocity is delayed under the visible-orbit condition at speeds greater than 0.5 Hz. Based on this, we suggest that using 3D visually presented guides can interfere with the learning processing that serves as a central component of cerebellar functions including motor learning and internal modeling.

## Supporting information

S1 Table Summary of statistical analysis results for Δ*θ*. (DOCX)

S2 Table Summary of statistical analysis results for Δ*ω*. (DOCX)
