# Supplementary material for: Visuomotor control of intermittent circular tracking movements with visually guided orbits in 3D VR environment
Source: PLoS One. 2021 May 27;16(5):e0251371. doi: 10.1371/journal.pone.0251371 (PMC8158929; doi:10.1371/journal.pone.0251371)
Supplement: S1 Table — (DOCX) [file pone.0251371.s001.docx]

S1 Table. Summary of the statistical analysis of Δ*θ*

| *Item* | *Variable* | *Test* | *Statistic* | *Confidence* |
| --- | --- | --- | --- | --- |
| A | Δ*θ* between the phase, speed, and orbit | Three-way repeated measures  ANOVA | phase:  Mauchly's Test*χ*^2^(0) = 0,  *p* = Nothing,*ε* = 1;  *F* (1,18) = 0.402;  speed:  Mauchly's Test*χ*^2^(2) = 29.20, *p* = 0,*ε* = 0.55;  *F* (1.1,19.78) = 64.11;  orbit:  Mauchly's Test*χ*^2^(0) = 0,  *p* = Nothing,*ε* = 1;  *F* (1,18) = 0.005;  phase$\times$orbit interaction:  Mauchly's Test*χ^2^(0) = 0,*  *p = Nothing,ε = 1;*  *F (1,18) = 4.85;*  speed$\times$orbit interaction:  Mauchly's Test*χ^2^(2) = 19.52, p = 0.0,ε = 0.594;*  *F (1.72,31.02) = 0.043*  phase$\times$speed$\times$orbit interaction:  Mauchly's Test*χ*^2^(2) = 10.55, *p* = 0.005,*ε* = 0.684;  *F* (1.368,24.619) = 5.35 | phase: *p* = 0.534, *partial η^2^* = 0.022    speed: *p* = 0, *partial η^2^* = 0.78  orbit: *p* = 0.944, *partial η^2^* = 0  phase$\times$orbit interaction:  *p* = 0.041, *partial η^2^* = 0.212  speed$\times$orbit interaction:  *p* = 0.94, *partial η^2^* = 0.002  phase$\times$speed$\times$orbit interaction:  *p* = 0.021, *partial η^2^* = 0.23 |
| B | Δ*θ* between the phase and orbit on *V(1)* | Two-way repeated measures  ANOVA | orbit:  Mauchly's Test*χ*^2^(0) = 0,  *p* = Nothing,*ε* = 1;  *F* (1,18) = 0.00;  phase:  Mauchly's Test*χ*^2^(0) = 0,  *p* = Nothing,*ε* = 1;  *F* (1,18) = 117.2;  orbit$\times$phase interaction:  Mauchly's Test*χ^2^(0) = 0,*  *p = Nothing,ε = 1;*  *F (1,18) = 4.268;* | orbit: *p* = 0.997, *partial η^2^* = 0.00  phase: *p* = 0.00, *partial η^2^* = 0.867  orbit $\times$ phase interaction:  *p* = 0.054, *partial η^2^* = 0.192 |
| C | Δ*θ* under the conditions of orbit$\times$phase on *V(1)* | Bonferroni-corrected pairwise comparisons | Orbit at *VIS*(1)  *t(18) = 1.57;*  Orbit at *VIS*(2)  *t(18) = 1.01;* | Orbit at *VIS*(1)  *p = 0.61,* *Cohen's d = 0.12;*  Orbit at *VIS*(2)  *p = 0.83,* *Cohen's d = 0.05;* |
| D | Δ*θ* between the phase, and orbit on *V(2)* | Two-way repeated measures  ANOVA | orbit:  Mauchly's Test*χ*^2^(0) = 0,  *p* = Nothing,*ε* = 1;  *F* (1,18) = 0.016;  phase:  Mauchly's Test*χ*^2^(0) = 0,  *p* = Nothing,*ε* = 1;  *F* (1,18) = 7.935  orbit$\times$phase interaction:  Mauchly's Test*χ^2^(0) = 0,*  *p = Nothing,ε = 1;*  *F (1,18) = 0.35;* | orbit: *p* = 0.899, *partial η^2^* = 0.001  phase: *p* = 0.011, *partial η^2^* = 0.306  orbit $\times$ phase interaction:  *p* = 0.561, *partial η^2^* = 0.019 |
| E | Δ*θ* under the conditions of orbit$\times$phase on *V(2)* | Bonferroni-corrected pairwise comparisons | Orbit at *VIS*(1)  *t(18) = 0.52;*  Orbit at *VIS*(2)  *t(18) = 0.21;* | Orbit at *VIS*(1)  *p = 0.13,* *Cohen's d = 0.36;*  Orbit at *VIS*(2)  *p = 0.33,* *Cohen's d = 0.23;* |
| F | Δ*θ* between the phase, and orbit on *V(3)* | Two-way repeated measures  ANOVA | orbit:  Mauchly's Test*χ*^2^(0) = 0,  *p* = Nothing,*ε* = 1;  *F* (1,18) = 0.038;  phase:  Mauchly's Test*χ*^2^(0) = 0,  *p* = Nothing,*ε* = 1;  *F* (1,18) = 9.220;  orbit$\times$phase interaction:  Mauchly's Test*χ^2^(0) = 0,*  *p = Nothing,ε = 1;*  *F (1,18) = 6.234;* | orbit: *p* = 0.848, *partial η^2^* = 0.002  phase: *p* = 0.007, *partial η^2^* = 0.339  orbit $\times$ phase interaction:  *p* = 0.022, *partial η^2^* = 0.257 |
| G | Δ*θ* under the conditions of orbit$\times$phase on *V(3)* | simple effects of orbit | Orbit at *VIS*(1)  *F* (1,18) = 2.338;  Orbit at *VIS*(2)  *F* (1,18) = 5.532; | Orbit at *VIS*(1)  *p* = 0.144, *partial η^2^* = 0.115  Orbit at *VIS*(2)  *p* = 0.03, *partial η^2^* = 0.235 |
| H | Δ*θ* under the conditions of orbit$\times$speed for phase | Bonferroni-corrected pairwise comparisons | *ORB*$(1)\times$*V*(1) for phase  *t(18) = 12.16;*  *ORB*$(1)\times$*V*(2) for phase  *t(18) = 1.89;*  *ORB*$(1)\times$*V*(3) for phase  *t(18) = 4.27;*  *ORB*$(2)\times$*V*(1) for phase  *t(18) = 5.1;*  *ORB*$(2)\times$*V*(2) for phase  *t(18) = 2.61;*  *ORB*$(2)\times$*V*(3) for phase  *t(18) = 0.86;* | *ORB*$(1)\times$*V*(1) for phase  *p = 0,* *Cohen's d = 2.79;*  *ORB*$(1)\times$*V*(2) for phase  *p = 0.07, Cohen's d= 0.43;*  *ORB*$(1)\times$*V*(3) for phase  *p = 0, Cohen's d = 0.98;*  *ORB*$(2)\times$*V*(1) for phase  *p = 0, Cohen's d= 1.17;*  *ORB*$(2)\times$*V*(2) for phase  *p = 0.02, Cohen's d = 0.6;*  *ORB*$(2)\times$*V*(3) for phase  *p = 0.4, Cohen's d= 0.2;* |
