# Supplementary material for: Visuomotor control of intermittent circular tracking movements with visually guided orbits in 3D VR environment
Source: PLoS One. 2021 May 27;16(5):e0251371. doi: 10.1371/journal.pone.0251371 (PMC8158929; doi:10.1371/journal.pone.0251371)
Supplement: S2 Table — (DOCX) [file pone.0251371.s002.docx]

S2 Table. Summary of the statistical analysis of Δ*ω*

| *Item* | *Variable* | *Test* | *Statistic* | *Confidence* |
| --- | --- | --- | --- | --- |
| A | Δ*ω* between the phase, speed and orbit | Three-way repeated measures  ANOVA | phase:  Mauchly's Test*χ*^2^(0) = 0,  *p* = Nothing,*ε* = 1;  *F* (1,18) = 78.94;  speed:  Mauchly's Test*χ*^2^(2) = 19.11, *p* = 0,*ε* = 0.60;  *F* (1.19,21.49) = 247.62;  orbit:  Mauchly's Test*χ*^2^(0) = 0,  *p* = Nothing,*ε* = 1;  *F* (1,18) = 29.61;  phase$\times$orbit interaction:  Mauchly's Test*χ*^2^(0) = 0,  *p* = Nothing,*ε* = 1;  *F* (1,18) = 9.52;  speed$\times$orbit interaction:  Mauchly's Test*χ*^2^(2) = 37.483, *p* = 0.0,*ε* = 0.529;  *F* (1.06,19.05) = 19.99  phase$\times$speed$\times$orbit interaction:  Mauchly's Test*χ*^2^(2) = 8.84, *p* = 0.01,*ε* = 0.711;  *F* (1.42,25.61) = 11.86 | phase: *p* = 0, *partial η^2^* = 0.81    speed: *p* = 0, *partial η^2^* = 0.932  orbit: *p* = 0, *partial η^2^* = 0.62  phase$\times$orbit interaction:  *p* = 0.006, *partial η^2^* = 0.346  speed$\times$orbit interaction:  *p* = 0.00, *partial η^2^* = 0.526  phase$\times$speed$\times$orbit interaction:  *p* = 0.001, *partial η^2^* = 0.397 |
| B | Δ*ω* between the phase, and orbit on *V(1)* | Two-way repeated measures  ANOVA | orbit:  Mauchly's Test*χ*^2^(0) = 0,  *p* = Nothing,*ε* = 1;  *F* (1,18) = 1.756;  phase:  Mauchly's Test*χ*^2^(0) = 0,  *p* = Nothing,*ε* = 1;  *F* (1,18) = 0.286;  orbit$\times$phase interaction:  Mauchly's Test*χ^2^(0) = 0,*  *p = Nothing,ε = 1;*  *F (1,18) = 0.447;* | orbit: *p* = 0.202, *partial η^2^* = 0.089  phase: *p* = 0.599, *partial η^2^* = 0.016  orbit $\times$ phase interaction:  *p* = 0.512, *partial η^2^* = 0.024 |
| C | Δ*ω* under the conditions of orbit$\times$phase on *V(1)* | Bonferroni-corrected pairwise comparisons | Orbit at *VIS*(1)  *t(18) = 0.83;*  Orbit at *VIS*(2)  *t(18) = 1.66;* | Orbit at *VIS*(1)  *p = 0.42,* *Cohen's d = 0.19;*  Orbit at *VIS*(2)  *p = 0.12,* *Cohen's d = 0.38;* |
| D | Δ*ω* between the phase, and orbit on *V(2)* | Two-way repeated measures  ANOVA | orbit:  Mauchly's Test*χ*^2^(0) = 0,  *p* = Nothing,*ε* = 1;  *F* (1,18) = 30.576;  phase:  Mauchly's Test*χ*^2^(0) = 0,  *p* = Nothing,*ε* = 1;  *F* (1,18) = 7.555;  orbit$\times$phase interaction:  Mauchly's Test*χ^2^(0) = 0,*  *p = Nothing,ε = 1;*  *F (1,18) = 0.484;* | orbit: *p* = 0.00, *partial η^2^* = 0.629  phase: *p* = 0.013, *partial η^2^* = 0.296  orbit $\times$ phase interaction:  *p* = 0.495, *partial η^2^* = 0.026 |
| E | Δ*ω* under the conditions of orbit$\times$phase on *V(2)* | Bonferroni-corrected pairwise comparisons | Orbit at *VIS*(1)  *t(18) = 4.05;*  Orbit at *VIS*(2)  *t(18) = 3.71;* | Orbit at *VIS*(1)  *p = 0.001,* *Cohen's d = 0.93;*  Orbit at *VIS*(2)  *p = 0.002,* *Cohen's d = 0.85;* |
| F | Δ*ω* between the phase, and orbit on *V(3)* | Two-way repeated measures  ANOVA | orbit:  Mauchly's Test*χ*^2^(0) = 0,  *p* = Nothing,*ε* = 1;  *F* (1,18) = 24.552;  phase:  Mauchly's Test*χ*^2^(0) = 0,  *p* = Nothing,*ε* = 1;  *F* (1,18) = 66.525;  orbit$\times$phase interaction:  Mauchly's Test*χ^2^(0) = 0,*  *p = Nothing,ε = 1;*  *F (1,18) = 14.636;* | orbit: *p* = 0.00, *partial η^2^* = 0.577  phase: *p* = 0.00, *partial η^2^* = 0.787  orbit $\times$ phase interaction:  *p* = 0.001, *partial η^2^* = 0.448 |
| G | Δ*ω* under the conditions of orbit$\times$phase on *V(3)* | simple effects of orbit | Orbit at *VIS*(1)  *F* (1,18) = 30.408;  Orbit at *VIS*(2)  *F* (1,18) = 11.688; | Orbit at *VIS*(1)  *p* = 0.000, *partial η^2^* = 0.628  Orbit at *VIS*(2)  *p* = 0.003, *partial η^2^* = 0.394 |
| H | Δ*ω* under the conditions of orbit$\times$speed for phase | Bonferroni-corrected pairwise comparisons | *ORB*$(1)\times$*V*(1) for phase  *t(18) = 0.03;*  *ORB*$(1)\times$*V*(2) for phase  *t(18) = 2.27;*  *ORB*$(1)\times$*V*(3) for phase  *t(18) = 8.4;*  *ORB*$(2)\times$*V*(1) for phase  *t(18) = 0.83;*  *ORB*$(2)\times$*V*(2) for phase  *t(18) = 1.9;*  *ORB*$(2)\times$*V*(3) for phase  *t(18) = 5.66;* | *ORB*$(1)\times$*V*(1) for phase  *p = 0.98, Cohen's d= 0.01;*  *ORB*$(1)\times$*V*(2) for phase  *p = 0.04, Cohen's d = 0.52;*  *ORB*$(1)\times$*V*(3) for phase  *p = 0, Cohen's d = 1.93;*  *ORB*$(2)\times$*V*(1) for phase  *p = 0.42, Cohen's d = 0.19;*  *ORB*$(2)\times$*V*(2) for phase  *p = 0.07, Cohen's d = 0.44;*  *ORB*$(2)\times$*V*(3) for phase  *p = 0, Cohen's d = 1.3;* |
